# Safety and Effectiveness of Roxadustat in Dialysis-Dependent Patients With Renal Anemia: A Hospital-Based Cohort Study

**DOI:** 10.7759/cureus.24427

**Published:** 2022-04-23

**Authors:** Oumarou Moussa, Cao Feng, Jian Xiong Wang, Xiao Sheng Li, Feng Xia Zhang, Xian Hu Tang

**Affiliations:** 1 Department of Nephrology, First Affiliated Hospital of Gannan Medical University, Ganzhou, CHN; 2 Department of Nephrology, Gannan Medical University, Ganzhou, CHN

**Keywords:** dialysis, effectiveness and safety, roxadustat, chronic kidney disease (ckd), on dialysis, renal anemia

## Abstract

Background

Randomized controlled trials (RCTs) have shown the efficacy and safety of Roxadustat and conclude that it has the potential to change the treatment for anemia associated with chronic kidney disease. However, the experience of its use from clinical perspectives post-approval is lacking.

Aim

Using a clinical practice context, this study aims to compare Roxadustat's effectiveness and tolerability with Erythropoietin (EPO) in patients with renal anemia undergoing dialysis.

Methods

We examined the clinical records of patients with a diagnosis of renal anemia on dialysis who were prescribed Roxadustat or Erythropoietin at the department of nephrology of the First Affiliated Hospital of Gannan Medical University from January 2021 to December 2021. Eligible hemodialysis (HD) or peritoneal dialysis (PD) patients with renal anemia, aged >18 or <75 years, without infection, active bleeding, and malignancy were recruited. These patients received Roxadustat or EPO based on the preferential prescription choice made by the nephrologists of the department. We retrospectively attempted to determine the treatment response measured by the change in hemoglobin rate, from baseline up to six months. We also explored the impact of various factors on the treatment response and reported adverse events.

Results

A total of 106 patients have been included in the final analysis, with 53 patients in each group. The mean age of the study group was 49.9 ± 13.6 years with the main Hb level at the baseline of 8.1 g/dL ± 1.23 g/dl. The gain of hemoglobin from the baseline averaged over six months was 2.2 ± 2.11 g/dl in the Roxadustat group compared with 1.1 ± 1.67 g/dL in the EPO group (*p=0.01*)*. *As compared to EPO,Roxadustat reduced the total cholesterol level by -0.59 ± 1.08 mmol/l versus -0.01 ± 1.28 mmol/l (*p=0012*) and the low-density lipoprotein (LDL) cholesterol by -0.48 ± 1.07 mmol/l versus -0.47 ± 1.05 (*p=0.017*) in the first three months. Associated factors with a non-response to treatment were age greater than 65 years (OR=6, 95% CI: 1.23-32.46, *p=0.02*), hypertension (OR=3.5, 95%CI: 0.89-13.25, *p=0.060*), and heart failure (OR=4.18, 95%CI:4.18 1.04-20.39, *p=0.040*). Although the proportion of hospitalization and infection was higher in the EPO group and the incidences of gastrointestinal symptoms (vomiting, nausea) and blood transfusions were higher in the Roxadustat group, there were no statistically significant differences.

Conclusion

Roxadustat improved hemoglobin compared to erythropoietin in patients undergoing dialysis with a safe profile but precautions should be taken for old patients with a cardiovascular medical history.

## Introduction

Chronic kidney disease (CKD) is a public health challenge with an increasing burden on mortality and morbidity worldwide [1]. It is a condition characterized by the progressive loss of kidney function which can lead ultimately to dialysis or renal transplantation. Global studies showed that CKD is continuously increasing all over the world due to the high prevalence of relevant risk factors such as diabetes and hypertension [1-2]. In China, the largest population in the world, the overall prevalence of chronic kidney disease was estimated at 10·8% and is predicted to increase in the next decades, especially among rural regions [2].

Anemia is one of the most serious and inevitable complications of the advanced stages of chronic kidney disease, with chronic inflammation, iron deficiency, and erythropoiesis as the major contributing factors [3]. Anemia caused by CKD is associated with cardiovascular disease, long-term hospitalization, cognitive impairment, and reduced quality of life [4]. International clinical practice guidelines recommend the treatment of anemia as an integral part of CKD management [5-6]. The actual treatment options for anemia in CKD patients are managed by erythropoiesis-stimulating agents (ESAs), iron therapy, and blood transfusion [6]. Nearly all patients in the advanced stage of CKD suffer from anemia, but many of them are not efficiently treated. Data from the Shanghai Renal Registry 2011 indicated that patients with CKD did not achieve the recommended hemoglobin (Hb) targets in China; only 39% to 46% of the patients who underwent hemodialysis achieved a target Hb of 10 to 12 g/dL (similar to the target recommended by the international guidelines [7]. Currently marketed ESAs are biological agents that must be administered intravenously (IV) or subcutaneously (SC). These administration routes are more complex than those required for oral medications, resulting in expensive costs, and in some centers, access to ESAs is an issue [8]. Additionally, there are more and more concerns about the safety of these anti-anemic agents, which may increase blood pressure and be associated with an increased risk of cardiovascular events if, inadvertently, hemoglobin levels get above target [7-8]. Clinical studies reported that some patients are hyporesponsive to ESAs and need even larger doses because of functional iron deficiency associated with inflammation but high doses of ESAs increase the risk of serious adverse events, including myocardial infarction, congestive heart failure, stroke, and death [9-10]. To these issues, a new therapeutic arsenal has been recently gaining attention. In late 2018, China became the first country to approve Roxadustat for chronic kidney disease patients with anemia [11]. This approval granted by the National Medical Products Administration is primally supported by Phase 3 clinical trial studies conducted in China and Japan, which reported the efficacy of Roxadustat in CKD patients on peritoneal dialysis and hemodialysis, and in non-dialysis-dependent chronic kidney disease patients by a statistically significant improvement in hemoglobin (Hb) levels [12].

Roxadustat is a first-in-class orally administered inhibitor of hypoxia-inducible factor (HIF) prolyl­ hydroxylase (PH), which increases hemoglobin levels with a mechanism of action that is different from that of erythropoiesis-stimulating agents [13]. Orally administrated three times a week, it increases Hb levels through a mechanism mimicking the natural effects of high altitude [14]. It has long been known that the production of Hb increases at high altitudes, and the factor induced by hypoxia encourages the production of red blood cells that carry hemoglobin [13-14]. By increasing the levels of the hypoxia-induced factor, Roxadustat raises Hb levels through the use of the body's own iron stores, resulting in the control of Hb without the need for iron supplements in non-iron deficient patients [9,14]. Many randomized controlled trials (RCTs) have shown the efficacy and safety of Roxadustat, concluding its future potential to change the treatment of anemia associated with chronic kidney disease, but the experience of its use from clinical perspectives post-approval is lacking. Therefore, we conducted this study to retrospectively observe the effect of Roxadustat on hemoglobin response where the drug was prescribed in clinical routine care, with the aim of describing the effectiveness and safety of Roxadustat for renal anemia in real-life situations. It was from continuous recruitment of chronic kidney disease stage 5 (CKD5) patients using Roxadustat over an observational period of time of one year in the Department of Nephrology of the First Affiliated Hospital of Gannan Medical University. Sociodemographic data, Roxadustat dosage, hemoglobin variations, and other clinical values were recorded. The primary outcome was the variation of hemoglobin from the initial prescription up to six months of use and the report of main side effects observed.

## Materials and methods

Ethics

This study was conducted with respect to the ethical principles for medical research involving human subjects, as stipulated by the Declaration of Helsinki. We got the approval of the medical committee of Gannan Medical University after a presentation of the study protocol in the presence of both hospital and school representatives. All variables and data included in our analysis were part of normal routine care and entirely follow standard hospital practices. There was in no way an add-on for specific purposes. The analysis included in the study looked at outcomes for a cohort of patients retrospectively treated in our department.

Patients

The data included in the study were non-identifiable and collected from medical files of patients with chronic kidney disease stage 5 undergoing maintenance hemodialysis (HD) or peritoneal dialysis diagnosed with renal anemia and receiving Roxadustat or EPO as treatment. All of them were regularly seen in the nephrology department of the First Affiliated Hospital of Gannan Medical University. The inclusion criteria were: CKD5 HD or PD patients followed in our department, diagnosed with renal anemia, aged between 18 and 75 years old, receiving Roxadustat or Erythropoietin, with a complete database. A complete database assumed the availability of a value for hemoglobin, albumin, lipid profile, C-reactive protein (CRP), ferritin, potassium, calcium, blood pressure, and the date of the first prescription of treatment for each patient. The mean of the two most recent hemoglobin values before the first prescription of hemoglobin or EPO was between 6.0 g/dL and <11 g/dL. The subject must not have received erythropoiesis-stimulating agents, blood transfusions, oral or intravenous (IV) iron therapy, or any other anemia-related treatment for at least three months before entering the study to prevent the possibility of these treatments affecting the outcome. Subjects with the following criteria were excluded: clinically significant infection or evidence of active underlying infection; active and severe liver disease; history of malignancy; blood transfusion within one month prior to the first prescription of the treatment; cause of anemia other than renal anemia; albuminemia less than 30 g/l; any active or recent history of blood loss; incomplete data at the baseline; discontinuation in follow-up; and the use of an iron supplement or other anemic treatment during the study period.

Study design

All patients admitted to the nephrology inpatient department were initially seen. Among them, those with the diagnosis of renal anemia were selected. After that, we applied our inclusion and exclusion criteria. Patients fulfilling the study requirements were selected. The collection of data includes a review of medical records where the medical history was saved (age, gender, blood pressure, past medical history, history of dialysis, concomitant drugs report, time and duration on dialysis, laboratory tests results, blood pressure, hospitalizations, and past medical history). All the clinical data collected at the baseline were screened and extracted from the medical database at the time points one, two, three, and six months. The starting dose of oral Roxadustat was based on the subject’s weight group and according to the hospital usage. The dose of both treatments basically was not subject to adaptation, discontinuation, or change during the study period. The patients in the EPO group were receiving 10000 unit/Sc injection once a week and in the Roxadustat group about 100 mg three times a week using the weight measurement at initial prescription. From the baseline, hemoglobin, white blood cells, red blood cells, platelets, uric acid, transaminases, lipid profile, potassium, calcium, phosphorus, vitamin B12, ferritin, folates, and CRP were assessed after one, two, three, and six months. At the end of the study, we considered the hemoglobin improvement rate to treatment as an achievement of hemoglobin level from the baseline of at least 0.75 g/dl. From the literature review, there are no unanimous criteria to define a successful hemoglobin response rate in dialysis dependents patients with anemia. In 2016, in a randomized 6 to 19 weeks open-label study comparing Roxadustat versus epoetin alfa for anemia in patients receiving maintenance hemodialysis, Provenzano et al. defined the primary endpoint in Part 1 (six-week cohorts), as the proportion of participants whose Hb levels did not decrease by >0.5 g/dL [15]. The hemoglobin response rate in another clinical trial was defined as an increase from the baseline of at least 1.0 g per deciliter in the hemoglobin level by Chen et al. [16]. In PYRENEES (Roxadustat in the Treatment of Anemia in End-Stage Renal Disease Patients on Stable Dialysis), a Phase 3 controlled study, where 836 patients were randomized, the non-inferiority of Roxadustat versus ESA was declared for the change in Hb levels from baseline to the average of weeks 28-36 since the lower bound of the 95% CI was higher than 0.75 g/dL [17]. In our study, we choose a change of 0.75 g/dl as the hemoglobin response rate.

Statistical analysis

Statistical analysis was performed using Epi Info version 3.5.4 (Centers for Disease Control and Prevention (CDC), Atlanta, Georgia), IBM SPSS version 20.0 (IBM Corp., Armonk, NY), and Microsoft Excel 2016 (Microsoft Corporation, Armonk, NY). Categorical variables are presented as rate (%) and continuous variables are expressed as mean ± SD. Means were expressed using the T-test. When there was equality between the variances, the analysis of variance (ANOVA) test was used. In the case of non-homogeneous variance, the Mann-Whitney Wilcoxon test was used for correction of the ANOVA test. The chi-square test or Fisher's exact test (when the chi-square was inappropriate) was used to evaluate the difference between proportions. A varied unified analysis was carried out using the odds ratio, then a logistic regression was carried out on the confounding factors to eliminate the confusion bias and identify the independent factors associated with the change in hemoglobin. We worked within a 95% confidence interval and a 5% margin of error α; α was set as 0.05.

## Results

Patient profiles and baseline characteristics

**Figure 1:**
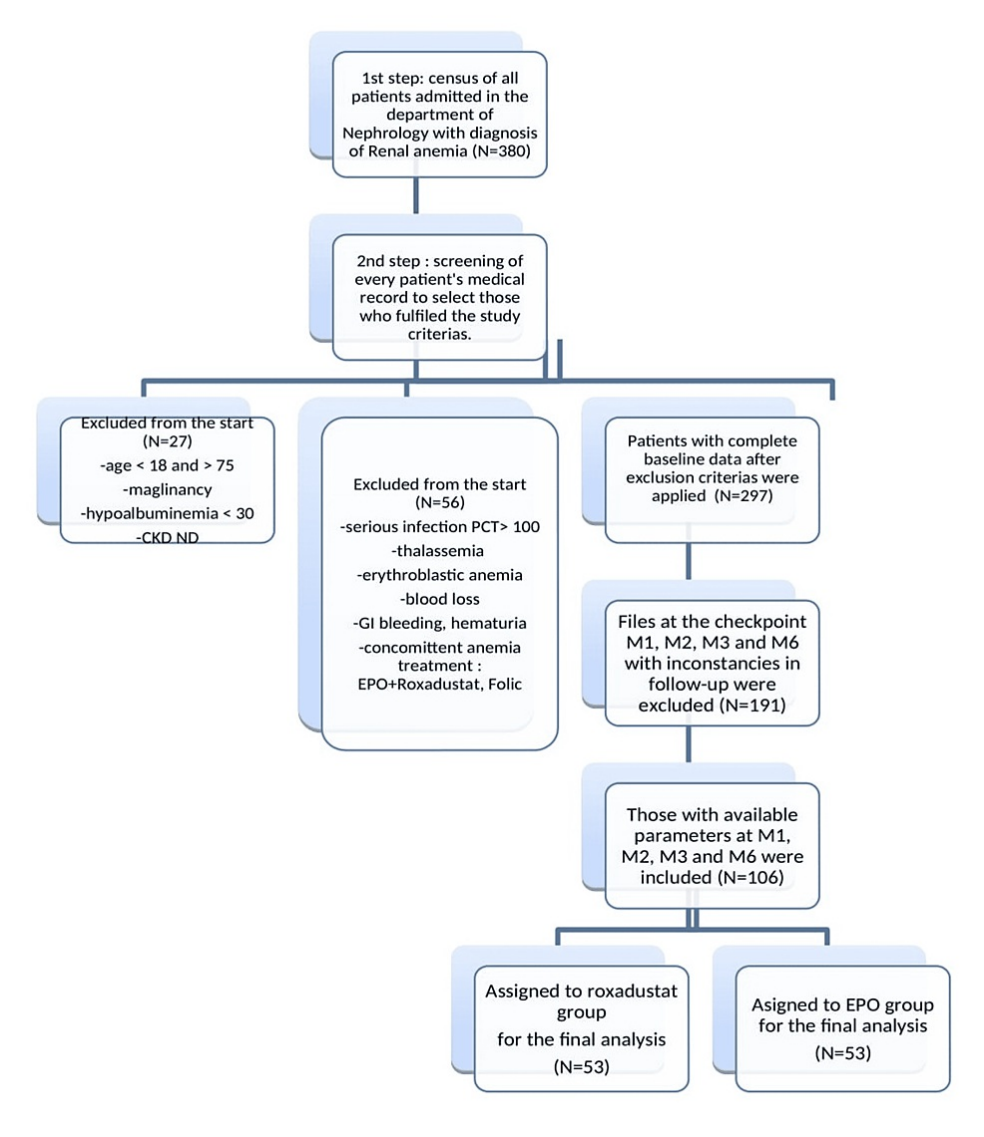
Flow chart of patients included in the study EPO: Erythropoietin. CKD ND: Non-Dialysis-Dependent Chronic Kidney Disease. PCT: Procalcitonin

After the screening of medical files, 380 patients were selected. Only a total of 106 patients with renal anemia, a complete database, and follow-up records were included in the final analysis (Figure 1). The patient profile and baseline characteristics have some similarities, with a notable difference between the two groups. The mean age of the study population is 49.9 ± 13.6 years old. Men were the most represented at 58.55%. Hypertension was the most observed associated condition (65%) followed by diabetes (31%), renal stones (21%), and glomerulonephritis (19%). Two-thirds of patients had heart insufficiency while more than half of them had secondary hyperthyroidism. In the Roxadustat group, 27 (45%) patients were on peritoneal dialysis. In the EPO group, HD was the leading modality for dialysis for 56.60% of patients. The anemic profile was normochromic normocytic anemia in this study (Table 1). The mean hemoglobin level at baseline was 8.17 ± 1.24 g/dl. Precisely, it was 8.39 ± 1.18 g/dl in the EPO group and 7.95 ± 1.26 g/dl in the Roxadustat group (P=0.069). The overall mean systolic blood pressure at the inclusion was 148 ± 23 mmHg. The mean baseline CRP was 17.2 mg/l. At baseline, the mean total cholesterol level was 4.04 ± 1.25 mmol/l in the Roxadustat group and 3.98 ± 1.20 mmol/l in the EPO group.

**Table 1:** Baseline characteristics of the study population ACEI: Angiotensin Conversing Enzyme Inhibitors. ARB: Angiotensin-Receptor Blockers. DBP: Diastolic Blood Pressure. EPO: Erythropoietin. HDL: High-Density Lipoprotein. IgA: Immunoglobulin A. LDL: Low-Density Lipoprotein. RBC: Red Blood Cells. Roxa: Roxadustat. PLT: Platelets. PPI: Proton Pump Inhibitor. PTH: Parathyroid Hormone. SBP: Systolic Blood Pressure. ULN: Upper Limit of Normal. WBC: White Blood Cells.

Characteristics	Total N=106	Roxa N =53	EPO N = 53
Age, mean (SD), years	49.9 (13.6)	47.75 (14.99)	52 (11.89)
Female n (%)	48 (45.28)	22 (41.50)	26 (41.50）
Male n (%)	58 (54.72)	31 (58.50)	27 (50.90)
Underlying Condition Requiring Dialysis N (%)			
Hypertension	69 (65)	31 (58.50)	38 (71.70)
Diabetes	33 (31)	17 (32.10)	16 (30.20%)
Gout	9 (8.5)	3 (5.70)	6 (11.30)
Stones	22 (21)	9 (17.0)	13 (24.50)
PCKD	1 (0.9)	1 (1.90)	00
Glomerulonephritis	20 (19)	15 (28.30)	5 (9.40)
Systemic disease	5 (4.7)	4 (7.50)	1 (1.90)
IgA nephropathy	9 (8.5)	4 (7.50)	5 (9.40)
Membranous nephropathy	1 (0.9)	1 (1.90)	00
Relevant Past Medical History N (%)			
Secondary hyperthyroidism	58 (55.00)	28 (52.80)	30 (56.60)
Previous stroke	8 (7.5)	6 (11.30)	2 (3.80)
Previous heart insufficiency	64 (60.37)	33 (62.30)	31 (58.50)
Gastritis	22 (21)	12 (22.60)	10 (18.90)
Hepatitis/cirrhosis	9 (8.5)	7 (13.20)	2 (3.80)
Atherosclerosis	13 (12)	6 (11.30)	7 (13.20)
Coronary artery disease	9 (8.5)	4 (7.50)	5 (9.40)
Dialysis			
Dialysis modality N (%)			
Hemodialysis (HD)	56 (52.83)	26 (49.05)	30 (56.60)
Peritoneal dialysis (PD)	44 (41.50)	24 (45.28)	20 (37.73)
HD+PD	6 (5.66)	3 (5.66)	3 (5.66)
Dialysis duration, mean (SD), months	15.32 (19.00)	16.62 (22.58)	14.02 (14.69)
< 6 months n (%)	50 (47.16)	29 (54.70)	21 (39.60)
Concomitant Drugs in Use N (%)			
Antihypertensive treatment	84 (79)	43 (81.13)	41 (77.35)
ACE inhibitors	7 (8.3)	4 (9.30)	3 (7.30)
ARB	34 (40)	19 (44.20)	15 (36.60)
Calcium channel blockers	74 (88)	41 (95.30)	33 (80.50)
Diuretics	6 (7.1)	2 (4.70)	4 (9.80)
Alpha-blockers	29 (35)	16 (37.20)	13 (31.70)
Insulin	25 (23.58)	14 (26.40)	11 (20.80)
Aspirin	6 (5.7)	2 (3.80)	4 (7.50)
Statin	22 (21)	12 (22.60)	10 (18.90)
Sevelamer	11 (10)	7 (13.20)	4 (7.50)
Calcium	51 (48)	27 (50.90)	24 (45.30)
Steroid	7 (6.6)	5 (9.40)	2 (3.80)
Cinacalcet	13 (12)	6 (11.32)	7 (13.20)
PPI	19 (17.92)	7 (13.20)	12 (22.64)
Chinese traditional medicine	60 (56.60)	39 (73.58)	33 (62.26)
Clinical Parameters			
Blood pressure			
SBP mean (SD), mmHg	147.85 (23.79)	149.40 (24.60)	146.30 (23.01)
DBP mean (SD), mmHg	86.51 (15.37)	86.81 (16.35)	86.21 (14.47)
WBC mean (SD)	7431 (4098)	7225 (2449)	7636 (5276)
RBC mean (SD)	2827 (581.13)	2710 (667.54)	2944 (456.92)
PLT mean (SD)	210.94 (87.89)	201.06 (66.75)	220.64 (102.91)
Hemoglobin mean (SD), g/l	8.17 (1.24)	7.95 (1.26)	8.39 (1.18)
Hemoglobin cohort n (%)			
6.0-9.0 g/l	73 (68.9)	42 (79.20)	30 (56.60)
9.0-11 g/l	33 (31.1)	11 (20.80)	23 (43.40)
Triglycerides, mean (SD), mmol/l	1.54 (1.23)	1.46 (1.27)	1.62 (1.20)
Total cholesterol, mean (SD), mmol/l	4.04 (1.25)	3.98 (1.20)	4.09 (1.32)
LDL cholesterol, mean (SD), mmol/l	1.82 (1.13)	1.30 (1.13)	2.34 (1.14)
HDL cholesterol, mean (SD), mmol/l	1.36 (1.81)	1.30 (1.66)	1.42 (1.96)
CRP mean, mg/l	17.24	15.77	18.74
AST, mean (SD), mmol/l	16.91 (16.81)	20.08 (15.08)	17.26 (12.47)
ALT, mean (SD), mmol/l	18.67 (13.84)	13.68 (13.40)	20.15 (19.23)
Hemocysteine, mean (SD), umol/l	22.47 (15.20)	25.47 (19.34)	19.47 (8.6)
Ferritin, mean, microgram/l	550 (109.8)	509 (68.1)	592 (14.03)
PTH mean, pg/ml	434	478	389
Albumin, mean (SD), g/l	37.67 (3.33)	34.77 (4.1)	40.57 (5.57)
Potassium, mean (SD), mmol/l	4.26 (0.85)	4.1 (0.86)	4.42 (0.85)
Calcium, mean (SD), mmol/l	1.94 (0.32)	1.91 (0.25)	1.96 (0.38)
Phosphorus, mean (SD), mmol/l	1.88 (0.67)	1.87 (0.71)	1.88 (0.64)
Uric acid mean (SD), umol/l	43.77 (12.83)	42.09 (12.69)	45.45 (12.87)
Folate, mean, nmol/l	9.91	9.50	10.33
Vitamin B12, mean pg/l	127.61	131.69	123.55

Hemoglobin change and hemoglobin improvement

**Figure 2:**
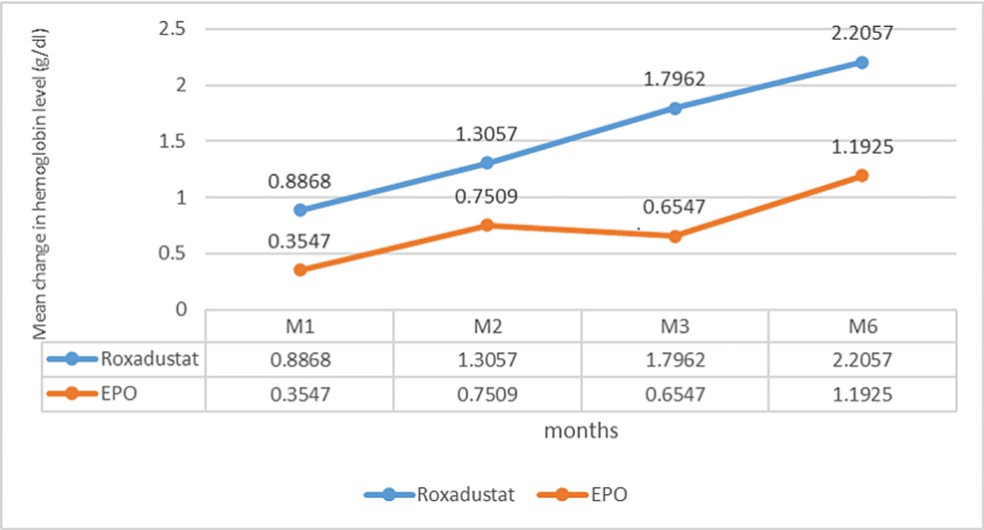
Estimated change in hemoglobin level

**Figure 3:**
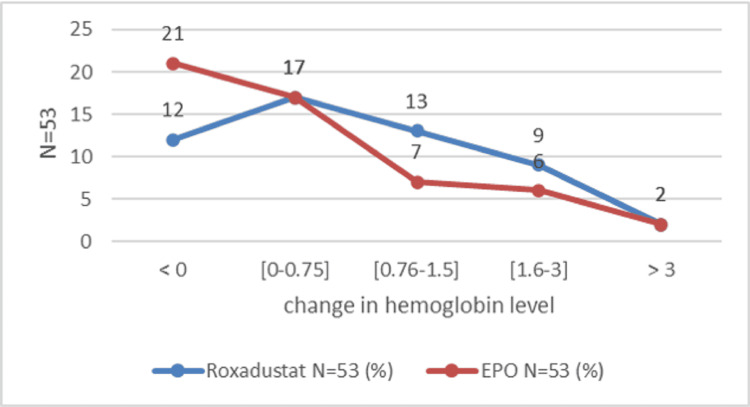
Proportion of patients who achieve the hemoglobin response rate after one month

**Figure 4:**
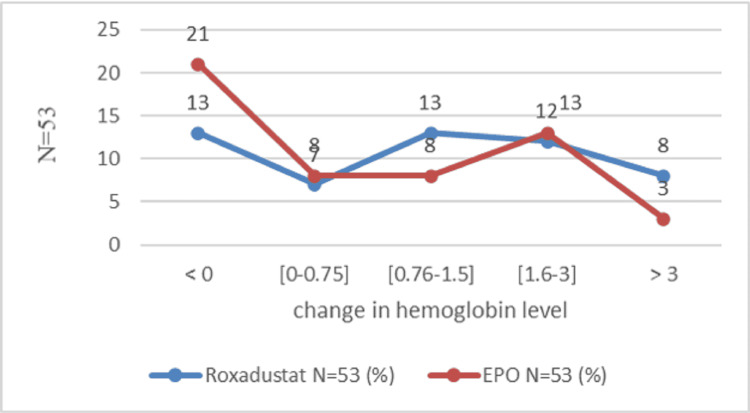
Proportion of patients who achieve the hemoglobin response rate after two months

**Figure 5:**
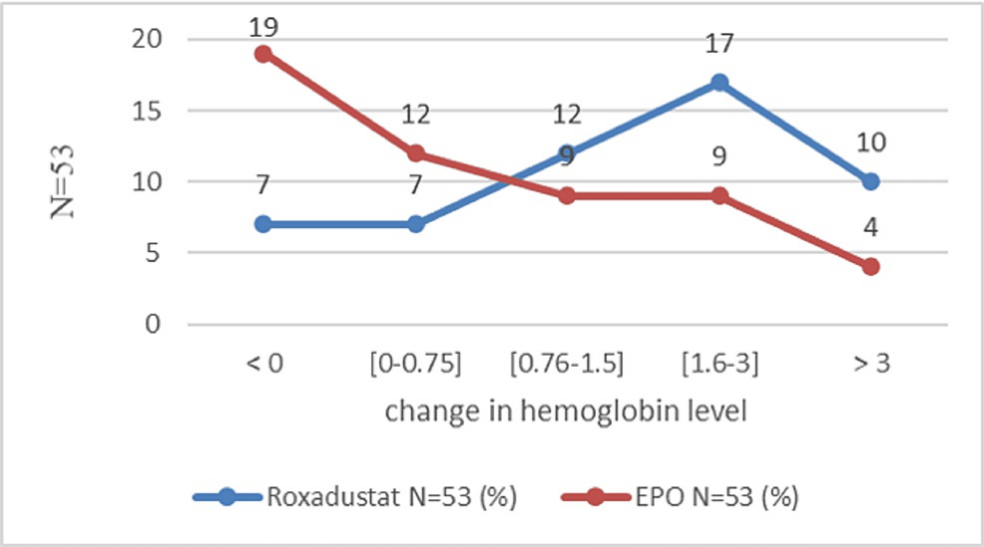
Proportion of patients who achieve the hemoglobin response rate after three months

**Figure 6:**
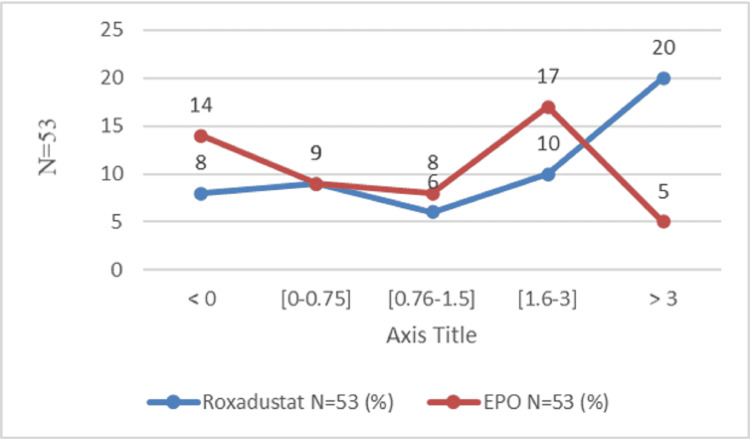
Proportion of patients who achieve the hemoglobin response after six months

The mean change in hemoglobin level was marked by an increase at the end of the study in both groups. At the end of the first month, the hemoglobin level increased by 0.88±1.23 g/dl in the Roxa group compared to 0.35 ± 1.28 g/dl in the EPO group (P=0.01). After the second month, the change in hemoglobin was 1.30 ± 1.76 g/dl in the Roxadustat group and 0.75 ± 1.46 g/dl in the EPO group (P=0.11). After three months, the hemoglobin level increased by 1.79 g/dl and 0.65 g/dl, respectively, in the Roxadustat and EPO groups (P=0.0015). After six months, the variation in hemoglobin level was 2.2 ± 2.11 g/dl in the Roxadustat group versus 1.1 ± 1.67 in the Epo (P=0.01) (Figure 2). The number of patients with a good therapeutic response is proportionally higher in the Roxadustat group than in the EPO group. An increase of at least 0.75 g/l defines an improvement in hemoglobin response [14]. This superiority is clear during the third month of treatment between the two groups: 63.90% (n=53) in the Roxadustat group versus 36.1% (n=53) in the EPO group (OR=3.93, 95% CI: 1.72-9.01, P=0.000) (Table 2). At the end of six months of treatment, a total of 66 patients out of 106 (62.3%) gained at least 0.75 g/dl in hemoglobin level: 54.5% (36 in 53) of patients in the Roxadustat group and 45.5% (30 in 53) of patients in the EPO group. After one month of treatment, Roxadustat failed to improve the hemoglobin level of 12 patients (Figure 3). The detailed results for the second month given are almost similar (Figure 4). After the third month, 37.73% (20 in 53) of patients in the Roxadustat group increased their baseline hemoglobin by 3 g/dl versus 9.43% (5 in 53) of patients in the EPO group (Figure 5). After six months, 26.14% (n=53) of patients in the EPO group versus 15% (n=53) in the Roxadustat group had no response (< 0 g/dl change in Hb from the baseline) (Figure 6).

**Table 2:** Proportion of patients with hemoglobin improvement rate over time EPO: Erythropoietin. Hb: Hemoglobin. OR: Odds Ratio. N: Effective Size. M: Month

	ROXADUSTAT	EPO	Total	OR (IC 95%)	P-value
Increase of Hb (g/dl)	n=53 (%)	n=53 (%)	N=106 (%)		
≥ 0.75 after M1	24 (61.5)	15 (38.5)	39 (36.8)	2.1 (0.93-4.75)	0.050
≥ 0.75 after M2	33 (57.9)	24 (42.1)	57 (53.8)	1.99 (0.91-4.36)	0.060
≥ 0.75 after M3	39 (63.9)	22 (36.1)	61 (57.5)	3.93 (1.72-9.01)	0.000
≥ 0.75 after M6	36 (54.5)	30 (45.5)	66 (62.3)	1.62 (0.73-3.62)	0.160

Effects on potassium and lipid profile

**Figure 7:**
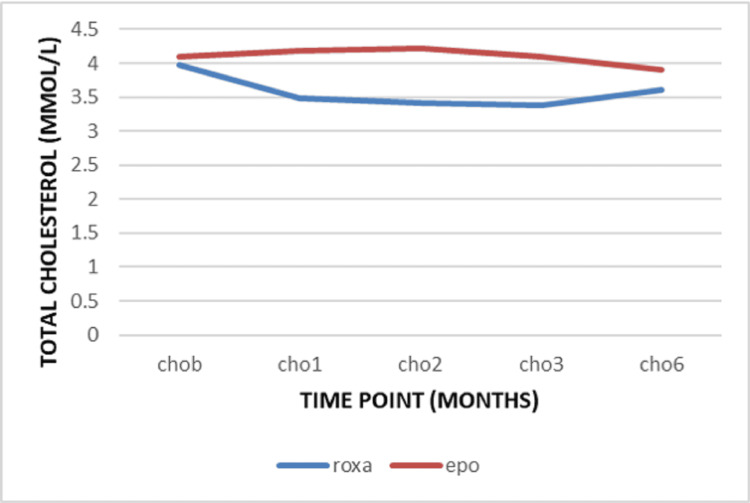
Variation of total cholesterol

**Figure 8:**
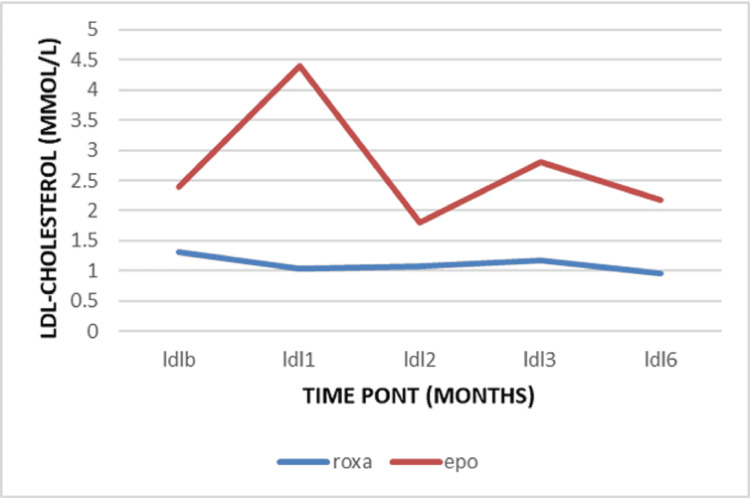
Variation of LDL cholesterol LDL: Low-Density Lipoprotein

The mean level of the serum potassium remains stable in the Roxadustat group with no remarkable change (Table 3). The mean triglycerides level at the baseline was 1.46 ± 1.27 mmol/l in the Roxadustat group and 1.62 ± 1.20 mmol/l in the EPO group. After one month, a decrease was observed in the Roxadustat group compared to the EPO group (-0.11 ± 1.23 mmol versus 0.07 ± 0.52) with no significance (*p=0.301*) (Table 4). At the beginning of the study, the mean total cholesterol level was 3.98 ± 1.20 mmol/l in the Roxadustat group and 4.09 ± 1.32 mmol/l in the EPO group. During the follow-up, the change was a decrease from the baseline (Figure 7). This reduction was statically more significant after three months of treatment by -0.59 ± 1.08 mmol/L in the Roxadustat group versus -0.01 ± 1.33 mmol/l in the EPO group (P=0.012). Meanwhile, the LDL cholesterol was 1.30 ± 1.13 mmol/l in the Roxadustat group versus 2.34 ± 1.14 mmol in the EPO group at baseline. There was a decrease from the baseline in LDL cholesterol level over time. This reduction was statistically greater after the first three months of treatment by -0.48 ± 1.07 mmol/l versus -0.48 ± 1.05 mmol/l (*p=0.017*) (Figure 8).

**Table 3:** Effect of Roxadustat on potassium *for Mean (SD) **for Welch two-sample t-test ***for potassium in mmol/l ****for variation represents the change or the difference in potassium level between the time point and the baseline

	Effects of Roxadustat		
Variable	EPO, N = 53*	Roxadustat, N = 53*	p-value**
Baseline			
Potassium***	4.4 (0.8)	4.1 (0.9)	0.049
After 1 Month			
Potassium	4.3 (0.8)	4.3 (0.8)	0.7
Variation****	-0.1 (0.9)	0.1 (0.9)	0.1
After 2 Months			
Potassium	4.3 (0.8)	4.2 (1.0)	0.6
Variation	-0.1 (0.9)	0.1 (1.2)	0.3
After 3 Months			
Potassium	4.3 (0.9)	4.2 (0.7)	0.5
Variation	-0.2 (1.0)	0.1 (1.1)	0.2
After 6 Months			
Potassium	4.4 (1.0)	4.1 (0.8)	0.2
Variation	-0.05 (0.1)	0.04 (1.01)	0.6

**Table 4:** Effects of Roxadustat on lipid profile ECB: Estimation Change from the Baseline. EPO: Erythropoietin. M: Month. Roxa: Roxadustat. SD: Standard Deviation

Group		baseline	M1	ECB1	p	M2	ECB2	p	M3	ECB3	p	M6	ECB6	p
Total Cholesterol (mmol/l)														
Roxa	Mean	3.98	3.49	-0.48	0.001	3.42	-0.55	0.001	3.38	-0.59	0.012	3.61	-0.398	0.465
	SD	1.2	1.19	0.83		1.07	0.94		1.13	1.08		1.10	1.20	
EPO	Mean	4.09	4.19	0.10		4.21	0.11		4.09	-0.01		3.95	-0.18	
	SD	1.32	1.34	0.83		1.20	1.13		1.04	1.28		1.09	1.33	
Triglycerides (mmol/l)														
Roxa	Mean	1.46	1.35	-0.11	0.301	1.56	0.09	0.413	1.59	0.13	0.243	1.60	0.13	0.471
	SD	1.27	0.84	1.23		1.70	2.08		1.80	1.42		1.74	1.29	
EPO	Mean	1.62	1.69	0.07		2.13	0.51		1.14	-0.20		1.54	-0.07	
	SD	1.20	1.23	0.52		3.45	0.02		1.18	1.07		1.30	1.29	
LDL Cholesterol (mmol/l)														
Roxa	Mean	1.30	1.04	-0.26	0.005	1.08	-0.22	0.012	1.17	-0.48	0.017	0.96	-0.46	0.80
	SD	1.13	1.01	0.87		1.04	1.01		1.05	1.07		0.97	0.85	
EPO	Mean	2.34	2.41	-0.39		2.38	-0.41		2.34	-0.48		2.18	-0.46	
	Sd	1.14	1.14	0.79		1.08	0.82		0.99	1.05		0.97	0.92	

Associated factors with no response to Roxadustat

Associated factors with a non-response to treatment were age greater than 65 years (OR=6, 95% CI: 1.23-32.46, *p=0.02*), hypertension (OR=3.5, 95%CI: 0.89-13.25, *p=0.060*), and heart failure (OR=4.18, 95%CI:4.18 1.04-20.39, *p=0.040*) (Table 5).

**Table 5:** Factors associated with no-response to Roxadustat HGB: Hemoglobin. OR: Odds Ratio. PPI: Proton Pump Inhibitor

		ROXADUSTAT												
		HGB<0.75		HGB≥0.75	Total				OR (CI 95%)			p-value		
AGE (years)		n=17 (%)		n=36 (%)	N=53 (%)									
< 25		1 (50)		1 (50)	2 (3.8)				2.19 (0.05-87.56)			0.540		
25 to 35 years		2 (16.7)		10 (83.3)	12 (22.6)				0.35 (0.05-1.69)			0.170		
35 to 45 years		2 (16.7)		10 (83.3)	12 (22.6)				0.35 (0.05-1.69)			0.170		
45 to 55 years		3 (37.5)		5 (62.5)	8 (15.1)				1.33 (0.23-6.55)			0.510		
55-65 years		3 (30)		7 (70)	10 (18.9)				0.89 (0.17-3.96)			0.600		
≥65 years		6 (66.7)		3 (33.3)	9 (17)				6 (1.23-32.46)			0.020		
GENDER														
Male		11 (35.5)		20 (64.5)	31 (58.5)				1.47 (0.44-5.12)			0.370		
Female		6 (27.3)		16 (72.7)	22 (41.5)				0.68 (0.2-2.28)			0.370		
DURATION OF DIALYSIS (months)														
0 to 6 months		8 (27.6)		21 (72.4)	29 (54.7)				0.63 (0.19-2.09)			0.320		
7 to 12 months		4 (44.4)		5 (55.6)	9 (17)				1.91 (0.39-8.63)			0.310		
13 to 36 months		3 (50)		3 (50)	6 (11.3)				2.36 (0.36-14.99)			0.290		
37 to 60 months		1 (16.7)		5 (83.3)	6 (11.3)				0.39 (0.02-3.15)			0.360		
More than 60 months		1 (33.3)		2 (66.7)	3 (5.7)				1.06 (0.03-14.83)			0.700		
INDICATION OF DIALYSIS														
Hypertension														
Yes	13 (41.9)		18 (58.1)			31 (58.5)				3.25 (0.89-13.25)			0.060	
No	4 (18.2)		18 (81.8)			22 (41.5)								
Diabetes														
Yes	6 (35.3)		11 (64.7)			17 (32.1)				1.24 (0.34-4.26)			0.480	
No	11 (30.6)		25 (69.4)			36 (67.9)								
Second Hyperthyroid														
Yes	8 (28.6)		20 (71.4)			28 (52.8)				0.71 (0.22-2.33)			0.390	
No	9 (36)		16 (64)			25 (47.2)								
Heart Insufficiency														
Yes	14 (42.4)		19 (57.6)			33 (62.3)				4.18 (1.04-20.39)			0.040	
No	3 (15)		17 (85)			20 (37.7)								
MODALITY OF DIALYSIS AND CONCOMITANT TREATMENT														
HD														
Yes	10 (33.3)			20 (66.7)				30 (56.6)			1.14 (0.35-3.84)		0.530	
No	7 (30.4)			16 (69.6)				23 (43.4)						
PD														
Yes	6 (22.2)			21 (77.8)				27 (50.9)			0.39 (0.11-1.31)		0.100	
No	11 (42.3)			15 (57.7)				26 (49.1)						
Prior Renal Anemia Treatment														
Yes	13 (38.2)			21 (61.8)				34 (64.2)			2.32 (0.63-9.56)		0.160	
No	4 (21.1)			15 (78.9)				19 (35.8)						
Anti-hypertensive														
Yes	13 (30.2)			30 (69.8)				43 (81.1)			0.65 (0.15-3.03)		0.400	
No	4 (40)			6 (60)				10 (18.9)						
Statin														
Yes	4 (33.3)			8 (66.7)				12 (22.6)			1.08 (0.24-4.27)		0.590	
No	13 (31.7)			28 (68.3)				41 (77.4)						
Sevelamer														
Yes	3 (42.9)			4 (57.1)				7 (13.2)			1.71 (0.28-9.27)		0.400	
No	14 (30.4)			32 (69.6)				46 (86.8)						
Calcium														
Yes	9 (33.3)			18 (66.7)				27 (50.9)			1.13 (0.35-3.69)		0.540	
No	8 (30.8)			18 (69.2)				26 (49.1)						
Cinacalcet														
Yes	1 (16.7)			5 (83.3)				6 (11.3)			0.39 (0.02-3.15)		0.360	
No	16 (34)			31 (66)				47 (88.7)						
PPI														
Yes	2 (28.6)			5 (71.4)				7 (13.2)			0.83 (0.1-4.74)		0.600	
No	15 (32.6)			31 (67.4)				46 (86.8)						
Chinese Traditional Medicine														
Yes	13 (33.3)			26 (66.7)				39 (73.6)			1.25 (0.33-5.36)		0.510	
No	4 (28.6)			10 (71.4)				14 (26.4)						

Safety analysis

The occurrence of vomiting was 3.77 % (2 in 53 patients) in the Roxa group. Overall, there were 24 cases of hospitalizations (4.5%) and 15 cases of infections (2.8%). Although the incidence rate of hospitalizations and infections was numerically greater in the EPO group than in the Roxadustat group, there was no statistically significant difference. The rescue therapy (for severe anemia or gastrointestinal (GI) bleeding) was reported in 9.43% (n=106) of the cohort. The standard group of EPO (3 in 53 patients) is less likely to experience the need for blood transfusion compared to the Roxadustat group (7/53 patients) but this founding is not statically significant (OR=0.39, 95% CI 0.96-1.61, *p=0.11*). No death was reported during the time of observation from the 106 patients who were included in the final analysis (Table 6).

**Table 6:** Frequency of probably related adverse effects *N=265, referer to the cumulative observations-times of side effects. From baseline after the initiation of the treatment to six months at the end of the treatment. It means 53 patients X 5 times. **N=53 for each group EPO: Erythropoietin. OR: Odds Ratio

SIDE EFFECTS	ROXADUSTAT N*=265 (%)	EPO N*=265 (%)	Total N*=530 (%)	OR (IC 95%)	p-value
Pulmonary Distress Syndrome					
Yes	1 (100)	0 (0)	1 (0.2)	/	0.500
No	264 (49.9)	265 (50.1)	529 (99.8)		
Upper Extremity Venous Thrombosis					
Yes	1 (100)	0 (0)	1 (0.2)	/	0.500
No	264 (49.9)	265 (50.1)	529 (99.8)		
Cardiovascular Disorder (AF, QRS Tachycardia)					
Yes	1 (33.3)	2 (66.7)	3 (0.6)	0.5 (0.02-6.59)	0.500
No	264 (50.1)	263 (49.9)	527 (99.4)		
Neurological Disorder (Insomnia)					
Yes	1 (100)	0 (0)	1 (0.2)	/	0.500
No	264 (49.9)	265 (50.1)	529 (99.8)		
Vomiting					
Yes	2 (100)	0 (0)	2 (0.4)	/	0.250
No	263 (49.8)	265 (50.2)	528 (99.6)		
Nausea					
Yes	2 (100)	0 (0)	2 (0.4)	/	0.250
No	262 (49.7)	265 (50.3)	527 (99.6)		
Gastrointestinal (Vomiting + Diarrhea)					
Yes	1 (100)	0 (0)	1 (0.2)	/	0.500
No	263 (50)	263 (50)	526 (99.8)		
Neurovascular Accident					
Yes	0 (0)	2 (100)	2 (0.4)	0 (0-3.47)	0.250
No	265 (50.2)	263 (49.8)	528 (99.6)		
Hospitalization During Follow Up					
Yes	11 (45.8)	13 (54.2)	24 (4.5)	0.84 (0.36-1.93)	0.420
No	254 (50.2)	252 (49.8)	506 (95.5)		
Infection During the Follow Up					
Yes	7 (46.7)	8 (53.3)	15 (2.8)	0.87 (0.3-2.51)	0.500
No	258 (50.1)	257 (49.9)	515 (97.2)		
Neurological Disorder (Dizziness)					
Yes	1 (50)	1 (50)	2 (0.4)	1 (0.03-39.14)	0.750
No	264 (50)	264 (50)	528 (99.6)		
Blood Transfusion During the Follow Up**					
Yes	7 (70.0)	3 (30.0)	10 (9.4)	0.39 (0.96-1.61)	0.113
No	46 (47.9)	50 (52.1)	96 (90.6)		

## Discussion

Overall, the main objective of our study was to report the effectiveness and tolerability of Roxadustat in the treatment of renal anemia in chronic dialysis (HD and PD) subjects over a period of six months by analyzing the change in hemoglobin level.

At the end of the study, we found that both Roxadustat and EPO increased baseline hemoglobin. This change was numerically greater in the Roxadustat group than in the EPO group. Roxadustat is a hypoxia-inducible-factor inhibitor that promotes red blood cell production through a mechanism of action similar to the natural way in which the human body responds to hypoxemia [18]. The findings of this study were in accordance with those from early studies where the capacity of Roxadustat to achieve hemoglobin change has been well-demonstrated. In 2018, Chen et al. were the first in China to conduct a clinical trial evaluating Roxadustat in Chinese patients undergoing long-term dialysis. He concluded that Roxadustat increased hemoglobin levels by 0.7 ± 1.1 g/dl compared to 0.5 ± 1.0 g/dl in the EA group and was non-inferior to epoetin alfa as therapy for renal anemia [19]. Provenzano et al. evaluated the efficacy of Roxadustat compared to epoetin alpha on incidents in patients with peritoneal dialysis or hemodialysis (for more than two weeks and less than four months with mean Hb ≤10.0 g/dL) and found similar results [20]. This hospital-based cohort study highlighted the fact that the modality for dialysis was not equally distributed in the two groups. PD was the main modality in the Roxadustat group compared to HD in the EPO group. PD patients have residual renal function and may possibly have elevated primary endogenous EPO levels. Additionally, patients on HD have more blood loss secondary to dialysis circuits and other vascular access complications resulting in a higher need for erythropoietin stimulants. In this setting of a retrospective study, this explanation may have probably played a substantial role in the final results. According to these findings, physicians tend to prescribe Roxadustat for PD patients and EPO for dialysis patients in clinical settings.

The significant presence of patients on peritoneal dialysis in the Roxadustat group could have affected the mean potassium level of the overall group and consequently explain why we do not find hyperkalemia as an adverse event. Also, hyperkalemia as an adverse effect of Roxadustat has been reported much more in studies that had a placebo as a comparator and in non-dialysis patients [21]. Patients without any replacement therapy (HD or PD) may experience metabolic acidosis or hyperkaliemia over time.

Even if the pathogenesis of dyslipidemia in CKD patients is still complex, it remains a traditional risk factor for cardiovascular events. As a HIF, Roxadustat may interfere with the overflow of intracellular cholesterol to apolipoprotein deficiency, resulting in a decrease in total cholesterol [22]. A beneficial effect of lowering total and low-density lipoprotein cholesterol can be efficacious in reducing coronary artery events in these patients [23].

Taken orally, Roxadustat is associated with more gastrointestinal side effects. Studies from Europe and Asia reported similar points in terms of serious adverse events [19,21,24]. In our study, patients on Roxadustat experienced more vomiting and nausea when compared to those on EPO. This can be an indicator of similar safety issues between the groups. One case of vascular access thrombosis was reported in the Roxadustat group. The reason may be the sudden variation of hemoglobin, the increase in the blood viscosity, and the inefficient response of iron compensation as stipulated in a recent publication [25].

Heart insufficiency, age more than 65 years old, and hypertension were the factors associated with a no-response (defined by <0.75 g/l) in the treatment with Roxadustat. They are all well-established as cardiovascular risk factors. Cardio-renal-anemia syndrome (CRAS) is a pathological triangle where heart failure, anemia, and chronic kidney disease are reciprocally aggravated by each other [26]. Anemia is an independent risk factor of mortality in patients with heart and kidney failure. The efficacy of Roxadustat to treat anemia in CKD patients was demonstrated in Phase 3 studies with more than 8,000 subjects worldwide. However, citing the safety surrounding Roxadustat, last year, the U.S. Food and Drug Administration (FDA) voted against the approval of Roxadustat in dialysis-dependent patients because of its potential thromboembolic and cardiovascular risks [27]. The clinical impacts of Roxadustat in CKD5 patients with heart failure have not yet been fully investigated thus far and remain unclear. Nevertheless, these risks are not exclusive to Roxadustat, as EPO is known to carry an increased risk of congestive heart failure as well as other thrombotic events [27-28]. In patients with heart failure, aggressive interventions (blood transfusion or ESA) to correct anemia are still controversial [28]. In our study, oral calcium acetate and Sevelamer were not found to be associated factors for non-response to Roxadustat as demonstrated elsewhere [29]. It was reported that calcium (prescribed to CKD patients for hyperphosphatemia) can reduce exposure to Roxadustat [30]. Nevertheless, in practice care patterns, various clinical factors may capture different outcomes.

It is imperative to consider some limitations of our study. Roxadustat patients tended to be on PD, whereas HD was most commonly used in the EPO group. The iron parameters, hepcidin and transferrin, were not routinely performed in our nephrology department. Also, we did not include results from heart ultrasound, and other cardiovascular parameters to fully evaluated the cardiovascular outcomes of our patients. Roxadustat was supposed to be more convenient for the patient (3 times a week, oral administration) and a tolerable option for the treatment of renal anemia, but its financial cost was not reported in this present study. The duration of six months may not be enough for an effective analysis of the safety of Roxadustat. A longer duration may be needed to determine if the effects of improvement in hemoglobin level we found from our results will remain constant. Despite knowing that there is a possible connection between the activation of the HIF pathway and the retina in our daily practice, we did not evaluate any patients for ophthalmologic problems. As a future goal, this is, for us, an opportunity to produce more original articles with subanalysis in the next submissions, exclusively focused on patients receiving HD or PD treatment.

## Conclusions

In this single-center study, the use of Roxadustat for the treatment of anemia in dialysis-dependent patients (HD and PD) was effective for increasing hemoglobin, with tolerable adverse side effects. More studies in clinical settings are necessary to carefully study the currently unclear effects and the long-term use benefits of Roxadustat.
